# Chondroitin Sulfate-Based MPDA@MnO_2_ Nanocomposite Hydrogels: A Smart Drug Delivery System with pH/ROS Responsiveness and Photothermal-Enhanced Therapeutic Effects

**DOI:** 10.3390/polym18111351

**Published:** 2026-05-29

**Authors:** Xu Wang, Qin Ding, Rui Ran, Qiangguo Chen, Xian Li, Xu Ye

**Affiliations:** 1 School of Materials and Chemistry, Southwest University of Science and Technology, Mianyang 621010, China; wangxuhh@outlook.com (X.W.);; 2 Hinova Pharmaceuticals Inc., Chengdu 610041, China; 3 School of Chemistry, Chemical Engineering and Life Sciences, Wuhan University of Technology, Wuhan 430070, China; 4 School of Continuing Education, Southwest University of Science and Technology, Mianyang 621010, China

**Keywords:** hydrogels, drug delivery, environmental stimulus response, ROS

## Abstract

Chronic wounds, particularly those complicated by infection, present significant challenges in clinical management. The microenvironment of these wounds is typically characterized by the accumulation of reactive oxygen species (ROS) and abnormal local pH levels, both of which impede the healing process. Baicalin (BA), a natural flavonoid, exhibits anti-inflammatory activity, ROS-scavenging capability, and pro-healing effects. In this study, hydrogels were synthesized through photoinitiated radical polymerization of methacrylic anhydride (MAA) and dopamine (DA)-modified chondroitin sulfate (ChSMA-DA), grafting degrees of MA and DA were 58%, 23%, MPDA@MnO_2_ nanoparticles (NPs), and methacrylated gelatin (GelMA). The gelation time, microtopography, swelling behavior, and water retention of the hydrogels were investigated, along with their degradation, rheological properties, and photothermal effects. The results indicate that swelling ratio (SR) and water retention (WR) of optimal HG-MPDA@MnO_2_-M sample were 5.7, 82.42%, exhibited responsive behavior upon weakly acidic environment with pH 6.5 and elevated ROS levels, and exhibited a stable photothermal effect (photothermal conversion efficiency was 22.7%) under 808 nm near-infrared (NIR) light. Following the incorporation of the drug model BA, the cumulative release percentage over 24 h under the combined stimulation of pH 6.5, 1 mmol·L^−1^ H_2_O_2_, and 808 nm NIR was 81.1%, significantly higher than either factor alone. These hydrogels show promise as an injectable dressing for chronic wounds, effectively integrating the internal microenvironment of the wound tissue with external NIR to modulate drug release.

## 1. Introduction

Chronic wounds, such as diabetic foot ulcers (DFUs) and pressure ulcers, pose significant challenges in clinical treatment [1]. Many advanced and intelligent hydrogels have been developed rapidly in medical Engineering, such as photothermal effect hydrogels, nanocomposite hydrogels [2], and DNA hydrogels [3], which are expected to provide solutions for chronic trauma. Most cutaneous wounds heal spontaneously within 1–2 weeks. However, the healing of chronic, deep, or burn wounds is often challenging due to bacterial infection. This infection causes significant tissue damage, enhanced inflammatory responses, and delays in wound repair [4]. The pH value of healthy skin is usually between 4.0 and 6.0 [5]. This acidity is central to barrier integrity, defense against pathogens, and maintaining a balanced skin microbiome [6]. The pH value of chronic wounds ranges from 7.1 to 8.9, and there are also clinical records of it reaching 6.4 and 9.0 [7]. During debridement and healing, the wound environment tends to shift toward acidity, and the pH varies depending on healing stage and infection status [8].

Upon skin injury, the pH of the wound microenvironment undergoes dynamic fluctuations [9]. During the inflammatory phase, bacterial infection, necrotic tissue degradation, and anaerobic metabolism caused by bacterial activity, tissue ischemia, and hypoxia produce organic acids such as lactic acid and acetic acid [10]. This process renders the microenvironment of the infected wound slightly acidic, with a pH of approximately 5.5 to 6.5. As inflammation subsides, new blood vessels form, and tissue regeneration begins, the pH of the wound gradually rises to a neutral or weakly alkaline level, around 7.15 to 8.9 [11,12]. This shift favors fibroblast proliferation and collagen deposition. However, in chronic wounds, impaired tissue repair capacity and persistent infection create a vicious cycle, trapping the wound in the acidic inflammatory stage [13]. Consequently, the pH remains weakly acidic for extended periods, preventing transition to the neutral proliferation stage and hindering the healing process. When the body experiences a pathological state, such as inflammation, tumors, or chronic diseases like hyperglycemia, reactive oxygen species (ROS) accumulate excessively in the local microenvironment [14], leading to oxidative stress [15]. Elevated ROS levels result in cellular damage, prolonged inflammation [16,17], and significantly impede wound healing [8]. The acidity and ROS levels in numerous solid tumors and inflammatory tissues are significantly higher than those in healthy tissues [18]. Conventional dressings and debridement techniques frequently encounter challenges in effectively addressing this complex and multifactorial pathological microenvironment.

The recurrence rate of diabetic foot ulcers (DFUs) within one year following successful healing reaches approximately 40% [19]. Over the past decade, nanosystem strategies tailored to the DFU microenvironment have been rapidly developed [20]. Nanoscale carriers can effectively penetrate biological barriers, including bacterial biofilms and necrotic tissues, thereby reaching deeper regions of ulcer wounds. This capability enhances drug bioavailability and prolongs therapeutic duration [21]. Additionally, nanocomposite hydrogels not only improve the mechanical strength and structural integrity of hydrogels [7] but also provide sensitive responses to external stimuli such as pH, light [22], and magnetic [23] fields. These features facilitate efficient drug loading [24], controllable release, and targeted delivery, making them highly suitable for wound dressing applications.

Photothermal therapy (PTT) is an innovative and controllable anti-tumor approach that eliminates tumors through the photothermal effect induced by near-infrared (NIR) irradiation [25]. Low-temperature PTT represents a safer alternative for cell therapy. Elevated local temperatures exceeding 50 °C can result in considerable cell necrosis and associated side effects. A temperature range of 42 to 45 °C may not be optimal, as cells exhibit heat tolerance [25]. In contrast, a temperature range of 45 to 50 °C may represent a more suitable PPT temperature range. Mesoporous polydopamine (MPDA) nanoparticles (NPs) exhibit excellent biocompatibility [26] and a high specific surface area [27], rendering them suitable carriers for drug delivery. The abundant catechol groups on the surface of MPDA confer a strong near-infrared photothermal response [28]. Upon exposure to 808 nm NIR irradiation, MPDA efficiently converts light energy into thermal energy, resulting in a mild photothermal effect that elevates the local tissue temperature [29,30]. This temperature increase can directly inhibit bacterial growth and simultaneously enhance the release of drug molecules encapsulated within the hydrogel network. Manganese dioxide (MnO_2_) degrades under weakly acidic conditions and participates in redox reactions in the presence of H_2_O_2_, catalyzing the decomposition of H_2_O_2_ to generate oxygen and water [31]. This property equips MnO_2_ with a dual capability to respond to the wound microenvironment: acid-triggered degradation enables controlled drug release, while its peroxidase-like activity scavenges excess H_2_O_2_, thereby reducing oxidative stress [32,33]. Furthermore, the oxygen produced helps alleviate tissue hypoxia, thereby facilitating the migration and proliferation of vascular endothelial cells [34]. The incorporation of MPDA and MnO_2_ enables hydrogel systems to adapt to the acidic and ROS-rich microenvironments while simultaneously leveraging external photothermal stimulation. The elevated temperature induced by PTT enhances the redox reaction between MnO_2_ and H_2_O_2_, leading to increased O_2_ production [35]. This, in turn, further accelerates drug release and enhances antibacterial efficacy via photothermal effects.

In response to the dynamic and complex microenvironment associated with chronic wounds, a hydrogel has been developed that intelligently adapts to microenvironmental changes while enabling active therapeutic regulation. Despite numerous studies on hydrogel-based drug delivery systems, integrated approaches combining nanotechnology, PTT, and conventional hydrogels remain relatively underexplored. We intentionally engineered a rationally integrated material system in which each component serves a distinct yet synergistic function. Chondroitin sulfate (ChS) was selected not only for its intrinsic anti-inflammatory and pro-angiogenic bioactivity but also for its high density of carboxyl and hydroxyl groups. These groups facilitate chemical modification and promote strong hydrogen-bonding and metal-coordination interactions with wound exudates and MnO_2_ surfaces. Gelatin, utilized as a mature hydrogel matrix, enhances crosslinking ability and mechanical stability, thereby improving the overall performance of the hydrogels. The dopamine-modified design aims to confer near-infrared (NIR) effects to the hydrogel, while MnO_2_ provides the foundational material for the hydrogel’s pH and ROS stimulation response. The preparation process for these materials involves direct light exposure and rapid solidification, minimizing excessive toxicity or side effects.

**Figure 1:**
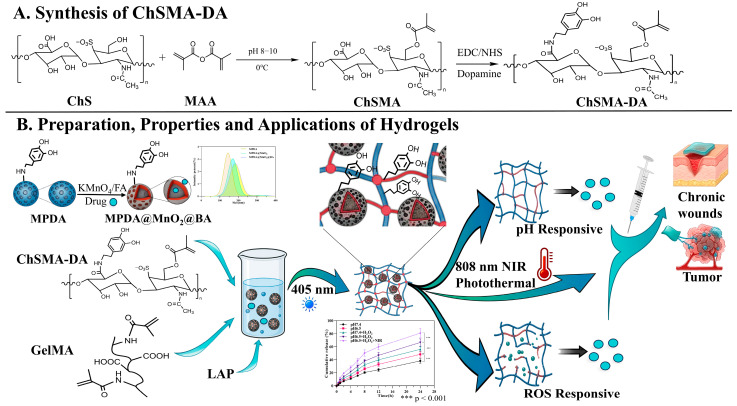
Schematic illustration of the construction route of hydrogel and the pH/ROS-responsive and photothermal-enhanced therapeutic effects. (**A**) Synthesis of ChSMA-DA, (**B**) Preparation, Properties, and Applications of Hydrogels.

The integration of ROS, pH, and NIR response mechanisms in a single injectable hydrogel is a nascent area of research with sparse literature, highlighting its innovative potential. Baicalin (BA) exhibits antioxidant, ROS scavenging, anti-inflammatory [36], anti-tumor [37], and promotes wound healing [38] properties. Its antioxidant and anti-inflammatory effects stem from scavenging ROS and enhancing antioxidant capacity by suppressing NF-κB activity and decreasing the expression of multiple inflammatory cytokines and chemokines [39]. It modulates inflammation, oxidative stress, angiogenesis, and infection control through multi-target mechanisms, demonstrating significant potential for chronic wound healing. As shown in Figure 1, this research integrates the photothermal effect of MPDA, the pH/ROS-responsive properties of MnO_2_, and drug delivery capabilities within nanomaterials to create an intelligent hydrogel system that can simultaneously respond to and synergistically interact with both “photothermal stimulation” and “weak acid/high ROS” environments. Chondroitin sulfate was modified with methylacrylamide to yield ChSMA. Through 1-(3-Dimethylaminopropyl)-3-ethylcarbodiimide (EDC)/*N*-Hydroxy succinimide (NHS) condensation and subsequent grafting with dopamine (DA), ChSMA-DA was synthesized, imparting both crosslinking capability and photothermal functionality. The introduction of methylacrylylated gelatin (GelMA) further enhanced the stability and mechanical properties of the hydrogel network. Utilizing a liquid-phase deposition/in-situ reduction strategy, the reducing properties of formamide (FA) and MPDA enabled the conversion of potassium permanganate to MnO_2_, which was then deposited on the surface and within the channels of MPDA to form MPDA@MnO_2_ NPs. These nanoparticles were dispersed in the ChSMA-DA and GelMA precursor solution, with lithium acylphosphinate (LAP) serving as the photoinitiator. Upon exposure to 405 nm irradiation, rapid photocrosslinking occurred, resulting in the formation of a stable three-dimensional hydrogel network that encapsulated the MPDA@MnO_2_ NPs. The physicochemical properties of the prepared MPDA@MnO_2_ NPs, including microstructure, particle size, and drug loading performance, were characterized. Additionally, the gelation time, microstructure, swelling properties, water retention, degradation behavior, photothermal effect, and drug release characteristics of the hydrogel were systematically investigated under various simulated physiological conditions to comprehensively assess the controllability and functionality of the materials.

## 2. Materials and Methods

### 2.1. Materials

Chondroitin sulfate (ChS) was obtained from Jiaxing Hengjie Biopharmaceutical Co., Ltd. (Shanghai, China) Methacrylic anhydride (MAA, 98%). Methacrylated gelatin (GelMA, MA degree 60%) was obtained from Aladdin Technology Co., Ltd. (Ha Noi, Vietnam) Dopamine hydrochloride (DA, ≥98%) and formamide (FA, ≥99.5%) were obtained from Adamas Life Sciences (Shanghai, China). 1-Ethyl-3-(3-dimethylaminopropyl) carbodiimide (EDC, ≥98%) and *N*-Hydroxysuccinimide (NHS, ≥98%) were obtained from Shanghai Adamas Reagent Co., Ltd. Potassium permanganate (≥99.5%) was obtained from Greagent (Edinburgh, UK). Lithium acylphosphinate (LAP, ≥95%) and hydrogen peroxide (H_2_O_2_, 3 wt.%) were obtained from Sigma-Aldrich (Burlington, MA, USA). Mesoporous polydopamine (MPDA) NPs were obtained from Jiangsu Xianfeng Nano Materials Technology Co., Ltd. (Jiangsu, China). Phosphate-buffered saline (PBS, pH 7.4) was prepared by dissolving 8.00 g NaCl, 0.20 g KCl, 1.42 g Na_2_HPO_4,_ and 0.24 g K_2_HPO_4_ in purified water to a final volume of 1000 mL. Baicalin (BA, ≥98%) was obtained from Shanghai Yuanye Biotechnology Co., Ltd. (Shanghai, China). Sodium hydroxide was obtained from Chengdu Kelong Chemical Reagent Factory (Chengdu, China). Dialysis bags (7–12 kDa) were obtained from Shanghai Yuanye Technology Co., Ltd. (Shanghai, China).

All other solvents and reagents were used as received without further purification.

### 2.2. Synthesis of MPDA@MnO_2_ NPs and Drug Load

MPDA@MnO_2_ NPs were synthesized via a liquid-phase deposition/in-situ reduction strategy, in which the reducing properties of MPDA NPs and FA were utilized to convert potassium permanganate into MnO_2_, followed by its deposition onto the surface and within the channels of MPDA NPs.

The MPDA NPs were dried prior to use, and 0.1 g of the resulting powder was dispersed in 50 mL of deionized water to obtain a concentration of 0.2% (*w*/*v*). The mixture was subjected to ultrasonic treatment for 10 to 20 min to ensure uniform dispersion. In a separate beaker, 0.1 g of potassium permanganate was dissolved in 50 mL of deionized water to obtain a concentration of 2.0 mg·mL^−1^. During continuous stirring of the MPDA NPs dispersion, 240 μL of formamide (FA) was added as a reducing agent and stabilizer. Subsequently, the potassium permanganate solution was slowly added dropwise to the MPDA NPs dispersion. The reaction mixture was stirred at room temperature for 12 h. The solution gradually changed from purple to brown or black, indicating the formation of manganese dioxide. The MPDA@MnO_2_ NPs were collected by centrifugation at 10,000 rpm for 10 min and washed several times with deionized water until the supernatant became colorless. Finally, the product was dried in an oven at 60 °C. The reaction can be represented as follows:

MPDA + 2KMnO_4_ + FA → MPDA@MnO_2_ ↓ + by-products (1)

For the loading of BA, 0.5% (*w*/*v*) BA solution was subsequently added dropwise to 10 mL of an aqueous dispersion of MPDA@MnO_2_ NPs at a concentration of 0.2% (*w*/*v*). The mixture was gently vortexed and incubated at 37 °C for 4 h in the dark to minimize photodegradation. Unbound BA was removed via centrifugation at 10,000 rpm for 10 min, and the resulting pellet was washed three times. The final BA-loaded nanoparticles (MPDA@MnO_2_@BA) were then re-dispersed in PBS and lyophilized for storage.

### 2.3. Characterization Test of MPDA@MnO_2_ NPs

#### 2.3.1. Element Analysis

X-ray photoelectron spectroscopy (XPS) was performed using a K-alpha spectrometer (Thermo Fisher, Waltham, MA, USA). A monochromatic X-ray source Al Kα (1486.6 eV) operating at 150 W (10 kV, 15 mA), was used for scanning. Elemental composition (C, N, O, and Mn) was further analyzed using energy-dispersive X-ray spectroscopy (EDS) with an EX-370 system (Hitachi High-Tech Corporation, Tokyo, Japan).

#### 2.3.2. Morphology Studies

The core-shell structure and particle size of MPDA@MnO_2_ NPs were characterized by transmission electron microscopy (TEM) using an HT7800 instrument (Hitachi High-Tech Corporation, Tokyo, Japan) at an accelerating voltage of 100 kV.

#### 2.3.3. Brunauer-Emmet-Teller (BET) Analysis

The specific surface area and pore structure of MPDA@MnO_2_ NPs were determined using a BET surface area analyzer (JW-BK200C, Beijing Jingwei Gaobo, Beijing, China). Nitrogen adsorption–desorption isotherms were measured at −196 °C. Nitrogen gas was gradually introduced to evaluate adsorption capacity at different pressures, followed by desorption measurements.

#### 2.3.4. Dynamic Light Scattering (DLS) Analysis

A nanoparticle size and zeta potential analyzer (Zetasizer Lab, Malvern, Worcestershire, UK) was employed to determine the hydrodynamic diameter and particle size distribution of the nanoparticles by dynamic light scattering (DLS). Approximately 1–2 mg of MPDA@MnO_2_ NPs powder was dispersed in 1 mL of water. The dispersion was sonicated in an ice bath. After sonication, the dispersion was allowed to stand for 5 to 10 min to sediment larger particles. The supernatant was subsequently transferred into a cuvette for DLS and zeta potential measurements.

#### 2.3.5. Drug Loading Studies

Drug loading capacity and encapsulation efficiency were determined using a UV–vis spectrophotometer (UV752 N, Shanghai Youke Instrument and Equipment Co., Ltd. Shanghai, China). A total of 10.0 mg of MPDA@MnO_2_ NPs was weighed and placed into centrifuge tubes. Then, 5.0 mg of baicalin was added along with an appropriate volume of solvent. The mixture was vortexed and ultrasonicated to enhance drug loading, followed by incubation at 25 °C for 24 h with shaking. The concentration of free BA in the supernatant was measured by UV spectroscopy. Drug loading capacity and encapsulation efficiency were calculated using the following equations:

Drug loading capacity = (*m*_l_/*m*_n_) × 100% (2)

Encapsulation rate = (*m*_l_/*m*_0_) × 100% (3)

where *m*_l_ is the mass of loaded BA, *m*_n_ is the mass of nanocarriers, and *m*_0_ is the initial mass of BA added.

### 2.4. Synthesis of ChSMA and ChSMA-DA

#### 2.4.1. Synthesis of ChSMA

ChS (2.5 g, 5 mmol) was dissolved in 200 mL of deionized water under stirring. Subsequently, 3 mL of methacrylic anhydride (MAA, 20 mmol) was added to the solution. The reaction was carried out in an ice-water bath at 0 °C, and the pH was maintained at 8–10 by dropwise addition of 1 mol·L^−1^ NaOH solution. The reaction proceeded for 24 h. The product was dialyzed (7–12 kDa) against deionized water for 3 days, with water changed three times daily to eliminate unreacted monomers and low molecular weight by-products. The dialyzed solution was frozen in liquid nitrogen and subsequently lyophilized in a freeze dryer to yield ChSMA, a pure white flocculent solid, which was stored at −20 °C for further use. The grafting degree of MA is calculated using the following Equation (4); a higher grafting degree of MA usually correlates with an accelerated cross-linking behavior of the hydrogel, resulting in increased cross-linking density, enhanced adhesion strength, and reduced network size.

Grafting degree of MA % = A_MA_/A_ChS_ × 100% (4)

where A_ChS_ is area of ChS methyl peak(–CH_3_), A_MA_ is area of MA methyl peak(–CH_3_).

#### 2.4.2. Synthesis of ChSMA-DA

ChSMA (0.54 g, 1 mmol) was dissolved in 10 mL of PBS buffer (pH 7.4) under stirring. EDC (0.24 g, 1.2 mmol) was added and stirred for 15 min, followed by the addition of NHS (0.12 g, 1 mmol) and stirring for another 15 min to activate the carboxyl group. Dopamine (0.15 g, 1 mmol) was then added, and the mixture was stirred for 24 h. After the reaction, unreacted dopamine and residual EDC/NHS were removed by dialysis using a 7–12 kDa dialysis bag against deionized water for 3 days, with the dialysis medium being replaced twice daily. The resulting solution was subsequently precipitated using a mixed 75% ethanol aqueous solvent, and the precipitate was collected. Finally, the ChSMA-DA powder was obtained by freeze-drying, and its structure was confirmed by NMR analysis. The grafting degree of dopamine was calculated using the following Equation (5); a greater grafting degree of DA is expected to theoretically lead to a more pronounced enhancement of the hydrogel’s photothermal effect.

Grafting degree of DA % = A_DA_/A_ChS_ × 100% (5)

where A_ChS_ is area of ChS methyl peak(-CH_3_), A_DA_ is the peak area of the aromatic protons on the benzene ring (Ar-H, Ar-H, Ar-H).

### 2.5. Preparation of Hydrogels

GelMA and ChSMA-DA were dissolved in PBS at 37 °C under continuous stirring. LAP (25 mg) was added and dissolved under dark conditions. To eliminate bubbles introduced during stirring and ultrasonication, the precursor solution was allowed to stand briefly to facilitate degassing, thereby preventing the formation of defects within the hydrogel during photo-crosslinking.

The ChSMA-DA/GelMA precursor was injected into the mold and subsequently irradiated with 405 nm light for 60 s, resulting in hydrogel formation. The gel was soaked in PBS at 37 °C for 30 min to reach equilibrium, followed by washing to remove residual unreacted monomers and initiator. The obtained hydrogels were designated as ChSMA-DA8/GelMA2, ChSMA-DA7/GelMA3, ChSMA-DA6/GelMA4 and ChSMA-DA5/GelMA5.

For NP-loaded hydrogels, the required amount of MPDA@MnO_2_ NPs or MPDA@MnO_2_@BA was weighed and added to the ChSMA-DA/GelMA precursor solution prepared in PBS at 37 °C. The mixture was ultrasonicated in an ice bath until a homogeneous dispersion without visible aggregation was achieved. The nanoparticle-loaded precursor was subsequently subjected to the same photo-crosslinking procedure. The resulting hydrogels were denoted as HG-MPDA@MnO_2_-N, HG-MPDA@MnO_2_-L, HG-MPDA@MnO_2_-M, and HG-MPDA@MnO_2_-H. All hydrogel formulations are summarized in Table 1.

FT-IR data were recorded using a Nicolet-7500 spectrometer (Thermo Electron Instruments Company, Waltham, MA, USA) in the spectral range of 4000–400 cm^−1^. The ^1^H-NMR spectra were recorded on a 600 MHz NMR spectrometer (Bruker Avance 600, Fällanden, Switzerland).

### 2.6. Gelation Time Studies of Hydrogels

Gelation time was assessed using the inverted test tube method. The prepared precursor solution and free radical initiator solution were added to a clean vial and mixed thoroughly. The vial was then inverted at regular intervals, and the gelation time was recorded as the point at which no flow was observed.

### 2.7. Microtopography Studies of Freeze-Dried Hydrogels

The microscopic morphology and pore structure of the freeze-dried hydrogel were characterized using scanning electron microscopy (SEM) (Ultra55, Carl Zeiss NTA GmbH, Jena, Germany). The samples were fixed onto the sample stage using conductive adhesive and subsequently sputter-coated with gold. The cross-sectional morphology was then observed under SEM at an accelerating voltage of 15 kV.

### 2.8. Swelling and Water Retention Studies of Hydrogels

The swelling ratio (SR) of the hydrogels was determined using a gravimetric method. Hydrogel samples were immersed in centrifuge tubes containing 5 mL of phosphate-buffered saline (PBS) and incubated in a water bath at 37 °C. At predetermined time intervals, the samples were removed, and the excess surface water was gently blotted using filter paper, followed by immediate weighing to obtain the wet weight. The SR values were calculated using:

SR = (*W*_s_ − *W*_0_)/*W*_0_ (6)

where *W*_s_ represents the mass of the swollen hydrogel at a given time point, and *W*_0_ is the mass of the initial dry hydrogel.

For water retention analysis, the dry hydrogel samples were immersed in PBS until swelling equilibrium was reached, then placed in an environment of constant temperature and humidity to record quality changes at a predetermined time. The water retention ratio (WR) was calculated by:

WR = (*W*_s_/*W*_0_) × 100% (7)

where *W*_s_ is the mass of the hydrogel at a specific time, and *W*_0_ is the initial dry mass.

### 2.9. Degradation Behavior Studies of Hydrogels

The degradation behavior of the hydrogels was examined using a weight loss method. Hydrogels with varying precursor concentrations were immersed in 5 mL of PBS solutions at pH 6.5, pH 7.4, and PBS containing 1 mmol·L^−1^ H_2_O_2_ to simulate physiological and oxidative environments. All experiments were conducted in triplicate. At predetermined time intervals, the samples were removed, rinsed, freeze-dried, and weighed to determine the remaining mass. The mass remaining (MR) was calculated using:

MR = (*W_t_*/*W*_0_) × 100% (8)

where *W*_0_ is the initial mass of the dry gel, and *W_t_* is the remaining mass after degradation at time *t*.

### 2.10. Rheological Studies of Hydrogels

Rheological properties of the hydrogels were tested using a rotational rheometer (DHR-1, TA Instruments, New Castle, DE, USA). Hydrogel samples (h = 0.5 cm, d = 2 cm) were prepared and loaded onto the rheometer platform. The storage modulus (*G*′) and loss modulus (*G*″) were recorded as functions of time, angular frequency, and strain at 37 °C.

### 2.11. Adhesion and Injectability Studies of Hydrogels

The adhesion and injectability properties were evaluated using an electronic universal testing machine (LST 01-1B, Shanghai Xihan Education and Science Equipment Co., Ltd. Shanghai, Chinia).

#### 2.11.1. Adhesion Studies

The adhesive properties were assessed using the lap shear test. The hydrogel was uniformly applied onto the overlapping area of the substrate, and the bonding area was measured. Given the photo-curable nature of the hydrogel, the adhesive layer was photopolymerized to form a stable interface. The prepared samples were fixed onto the upper and lower clamps of the testing machine. A tensile force was applied at a constant rate parallel to the bonding surface until failure occurred, and the maximum force was recorded. The lap shear strength (LSS) was calculated using:

LSS (kPa) = *F*/*A* (9)

where *F* is maximum tensile force (kN), and *A* is bonding area (m^2^).

#### 2.11.2. Injectability Studies

Injection properties were evaluated by loading the hydrogel into a 1 mL syringe. The syringe was mounted onto the testing apparatus, with the plunger aligned with a force sensor. The piston was advanced at a constant rate to extrude the hydrogel, while the applied force was continuously recorded throughout the extrusion process.

### 2.12. Photothermal Studies of Hydrogels

#### 2.12.1. Photothermal Heating Studies

Hydrogel samples (10 wt%) were dispersed in a quartz cuvette. The samples were irradiated using an 808 nm near-infrared laser at a power density of 1.0 W/cm^2^ for 10 min. The temperature was continuously monitored, and the temperature–time curves were recorded to evaluate the photothermal conversion performance.

#### 2.12.2. Photothermal Stability Studies (Laser on/off Cycle)

Hydrogel samples (10 wt%) were dispersed in a quartz cuvette and subjected to irradiation with an 808 nm NIR laser. Five on/off irradiation cycles were performed at 5 min intervals. The initial temperature was maintained at 25 ± 1 °C, and the temperature variation was recorded throughout the cycling process. The photothermal conversion efficiency (η) [40] was calculated using Equations (10) and (11):

*τ*s = mC/*h*S (10)

(11) $$\eta =\frac{hS\left(Tmax-Troom\right)-hS(Twater,max-Troom)}{I(1-10^{-A})}$$

where *τ*s is the thermal time constant, *h* is the heat transfer coefficient, and S refers to the area of the container. m = 1 g is the mass of the HG-MPDA@MnO_2_-M solution, C is the heat capacity (Cwater = 4.2 J/(g·℃)). *τ*s was calculated by Fitting curves of the time logarithm of the temperature during the cooling process, and the value of *h*S was calculated using Equation (10). In Equation (11), A is the absorbance of samples (500 μg·mL^−1^) at 808 nm, *I* is the NIR laser power, and T is temperature.

### 2.13. In Vitro Drug Loading and Release Studies of Hydrogels

BA was co-incubated with MPDA@MnO_2_ NPs in an appropriate solvent system, allowing the drug to be adsorbed onto the surface and within the pores of the nanoparticles, forming MPDA@MnO_2_@BA. The drug-loaded nanoparticles were subsequently incorporated into the ChSMA-DA/GelMA precursor solution, followed by photo-crosslinking to obtain drug-loaded hydrogels.

In vitro experiments were conducted to characterize the drug release behavior, leveraging the multi-factor environmental stimulus response characteristics of hydrogels. Hydrogel samples (HG-MPDA@MnO_2_-M@BA) were immersed in media with different conditions, including pH 7.4, pH 6.5, pH 7.4 + H_2_O_2_, and pH 6.5 + H_2_O_2_, pH 6.5 + H_2_O_2_ + NIR, and incubated at 37 °C under shaking conditions. pH 6.5 and 7.4 are PBS buffer solutions to simulate weak acid and normal tissue environments, 1 mol·L^−1^ H_2_O_2_ simulates high-level ROS environments, and 808 nm NIR (irradiation intensity of 1.0 W/cm^2^) simulates in vitro irradiation. At predetermined time intervals, 100 µL of the solution was withdrawn and diluted with 5 mL of PBS buffer solution. The absorbance was measured at 278 nm using a UV-Vis spectrophotometer (Thermo Fisher, Waltham, MA, USA). An equal volume of fresh medium was added to maintain a constant volume. All experiments were conducted in triplicate. The cumulative release of BA was calculated using Equations (12) and (13).

Cumulative release (%) = (*M_t_*/*M*_0_) × 100% (12)

(13) $$M_{t}=C_{t}\times 5+\sum_{1}^{t-1}C_{t-1}\times 0.1$$

where *M*_0_ is the mass of initial BA loading, *M_t_* is the cumulative release mass of BA at time *t*, and *C_t_* is the concentration of cumulative release BA at time *t*.

### 2.14. Kinetic Modeling of Baicalin Release Studies

The release of BA in pH 6.5 + H_2_O_2_ + NIR stimuli was described using various common drug release models, including Zero-order, First-order, Higuchi, Korsmeyer–Peppas, Hixson–Crowell, and Baker–Lonsdale models [41]. Mathematical models were simulated using Equations (14)–(19).

Zero-order                    *M_t_* = k_0_ × *t*   (14)

First-order               *M_t_* = 100 × [1 − e^(−k^_1_^×*t*)^]  (15)

Higuchi                     *M_t_* = k_H_ × *t*^0.5^  (16)

Hixson–Crowell         *M_t_* = 100 × [1 − (1 − k_HC_ × *t*)^3^]  (17)

Korsmeyer–Peppas                 *M_t_* = k_KP_ × *t^n^*  (18)

(19) $$\begin{array}{cc} \mathrm{Baker}–\mathrm{Lonsdale}\;\;\;\;\; & \frac{3}{2}\times [1-(1-M_{t}/100{)}^{\left(2/3\right)}]-M_{t}/100=k_{\mathrm{BL}}\times t \end{array}$$

where *M_t_* is cumulative release at time *t*, *t* is time (min), k_0_, k_1_, k_H_, k_HC_, *n*, k_KP_, and K_BL_ are constants.

### 2.15. Statistical Analysis

All data were plotted and analyzed using GraphPad Prism 8.3.0 software. Two-way analysis of variance (ANOVA) was performed for statistical comparisons, followed by Tukey’s post hoc test for multiple group analysis. Statistical significance was defined as * *p* < 0.05, ** *p* < 0.01, *** *p* < 0.001.

## 3. Results and Discussion

### 3.1. Characterization of MPDA@MnO_2_ NPs

#### 3.1.1. Element Results

**Figure 2:**
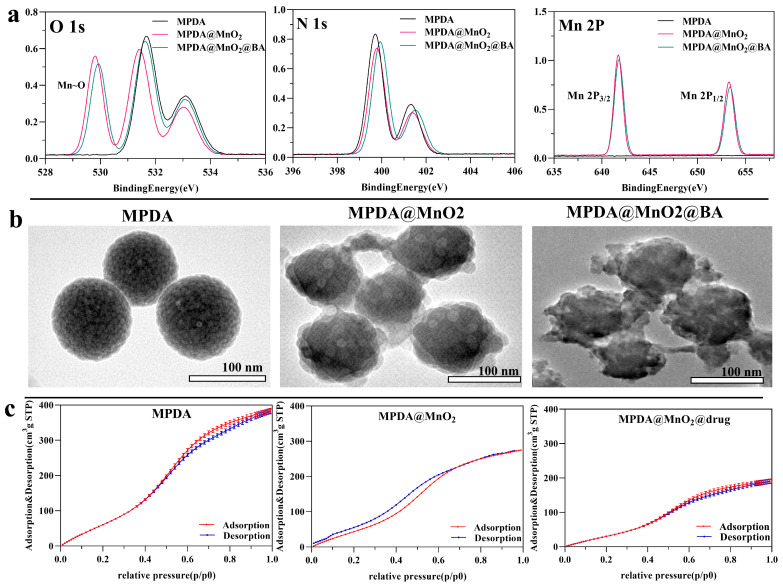
Characterization of MPDA NPs, MPDA@MnO_2_ NPs, and MPDA@MnO_2_@BA. (**a**) XPS spectra of O 1s, N 1s, Mn 2p, (**b**) TEM images, (**c**) Adsorption and desorption curves.

The XPS results (Figure 2a) confirm the successful synthesis of MPDA@MnO_2_ NPs and MPDA@MnO_2_@BA. Characteristic peaks for Mn 2p_3/2_ and Mn 2p_1/2_ were observed at 642 eV and 653 eV, respectively, in both MPDA@MnO_2_ NPs and MPDA@MnO_2_@BA, indicating that manganese is present on the particle surface in a stable oxidation state. The N 1s signals primarily appear at 400 eV and 401 eV, corresponding to pyrrole/amine nitrogen and protonated nitrogen, respectively, thereby confirming the preservation of the polydopamine (PDA) framework. The O 1s at 530 eV is attributed to Mn–O, while the 532 eV corresponds to oxygen-containing structures such as C=O and C–O.

#### 3.1.2. Morphology Results

TEM images (Figure 2b) illustrate a well-defined stepwise construction of the MPDA NPs-based drug delivery system. Initially, bare MPDA NPs particles display a highly regular spherical morphology with distinct boundaries; quantitative measurements show an average core diameter of 91.92 ± 7.08 nm and a total particle diameter of 92.20 ± 7.10 nm, indicating minimal shell thickness and confirming the absence of an existing surface layer. Upon MnO_2_ coating, the spherical shape remains intact, but an electron-dense outer layer becomes visible in TEM images; consequently, the total particle diameter increases to 110.39 ± 7.32 nm, with an average MnO_2_ shell thickness of 8.82 ± 1.61 nm, thereby demonstrating the successful formation of a uniform, conformal inorganic shell. Loading BA leads to a statistically significant increase in total diameter to 116.10 ± 8.14 nm, with the measured shell thickness reaching 10.82 ± 1.65 nm, supporting BA integration into or onto the MnO_2_ layer, thereby thickening the outer functional domain. These TEM findings offer direct structural evidence of the rational assembly of the system in three sequential, morphologically identifiable stages: (i) synthesis of monodisperse mesoporous MPDA NPs cores, (ii) controlled in situ growth of a uniform MnO_2_ responsive shell, and (iii) efficient loading of the therapeutic payload (BA), all while maintaining colloidal integrity and spherical symmetry.

#### 3.1.3. BET Results and Drug Loading Ability

BET nitrogen adsorption-desorption (Figure 2c) analysis systematically confirmed the evolution of the mesoporous structure of MPDA NPs and its composites. Following MnO_2_ coating and BA loading, the specific surface area (SA), pore volume (PV), and average pore diameter of the material exhibited a significant and progressive decline. This trend strongly supports the orderly occupation mechanism of mesoporous channels by shell construction and drug loading. The original MPDA NPs displayed typical mesoporous characteristics, with a specific surface area of 598.49 ± 17.38 m^2^·g^−1^, a pore volume of 0.739 ± 0.009 cm^3^·g^−1^, and an average pore diameter of 12.05 ± 0.69 nm. Following in-situ coating with MnO_2_, the surface area (SA) decreased to 432.76 ± 5.23 m^2^·g^−1^, the pore volume (PV) decreased to 0.530 cm^3^·g^−1^, and the average pore size decreased to 8.62 ± 0.20 nm. After further loading with baicalin, these values decreased to 279.79 ± 10.54 m^2^·g^−1^, 0.317 ± 0.013 cm^3^·g^−1^, and 7.68 ± 0.07 nm, respectively. This attenuation trend is associated with the stepwise construction of the material. MnO_2_ NPs preferentially enter and partially fill the inner walls of the MPDA NPs channels, forming a dense inorganic shell layer. Subsequently, BA molecules are anchored to the surface of MnO_2_ and the remaining pore openings through physical adsorption or weak interactions, resulting in secondary spatial confinement.

The adsorption-desorption isotherms of all nanoparticle samples are classified as type IV according to IUPAC standards [42]. The MPDA NPs exhibits a distinct H1 lag loop in the *p*/*p*_0_ range of 0.5–0.9, indicating its high connectivity and uniform cylindrical mesoporous structure. The hysteresis loop of MPDA@MnO_2_ NPs shifts leftward to *p*/*p*_0_ = 0–0.65, with the adsorption branch in the high *p*/*p*_0_ region rising gradually. This change reflects a contraction of the channel inlet and a reduction in internal permeability. At the MPDA@MnO_2_@BA stage, BA introduces spatial hindrance at the pore opening, resulting in a sharp decline in adsorption capacity at very low *p*/*p*_0_ (<0.1), potentially leading to the formation of salt-like or spatially hindered pore entrances. In summary, the steric hindrance or shielding effect at the active sites, which arises from the modification and loading process, consistently reduces the overall adsorption performance. The changes in specific surface area and pore structure suggest that this carrier possesses significant structural adaptability and loading capacity for BA.

#### 3.1.4. EDS and DLS Results

**Figure 3:**
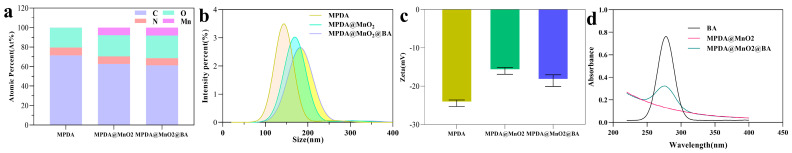
Characterization of MPDA NPs, MPDA@MnO_2_ NPs, and MPDA@MnO_2_@BA. (**a**) EDS, (**b**) DLS curves, (**c**) Zeta potential, (**d**) UV spectra of drug BA-loaded.

EDS (Figure 3a) reveals a relatively uniform distribution of carbon and nitrogen elements within the internal regions of the particles, while the Mn signal further corroborates the presence of the MnO_2_ shell layer based on its spatial distribution.

DLS and zeta potential analyses collectively indicate that sequential structural modifications involving MnO_2_ shell coating and BA loading systematically modify the hydrodynamic size, polydispersity, and surface charge of MPDA NPs. Specifically, DLS data (Figure 3b) show a gradual increase in the Z-average hydrodynamic diameter: from 148.44 ± 2.08 nm for pristine MPDA NPs (PDI = 0.124 ± 0.013) to 172.38 ± 0.48 nm after MnO_2_ coating (PDI = 0.165 ± 0.011), and further to 185.26 ± 2.09 nm following BA loading (PDI = 0.180 ± 0.006). This gradual increase in particle size reflects the combined impact of the inorganic MnO_2_ shell and the adsorbed/encapsulated drug molecules on the hydration layer, consistent with the core–shell thickness observed in TEM images. Importantly, all PDI values remain below 0.20, indicating a narrow size distribution and good colloidal uniformity throughout the modification process. Simultaneously, zeta potential measurements (Figure 3c) demonstrate that the MPDA NPs surface maintains a consistently negative charge (−24.43 ± 0.83 mV), which partially neutralizes after MnO_2_ coating (−16.00 ± 0.89 mV) and undergoes a slight shift after BA loading (−18.53 ± 1.52 mV). The persistent negative surface potential confirms the continued electrostatic stabilization and further validates the successful interfacial engineering: the decrease in absolute zeta potential upon MnO_2_ deposition suggests shielding or partial charge compensation by the oxide layer, while the subsequent change after BA loading indicates molecular interactions (such as hydrogen bonding or π–π stacking) between baicalin and the MnO_2_-coated surface.

#### 3.1.5. Drug Loading Analysis

The BA loading capacity and encapsulation efficiency of MPDA@MnO_2_ NPs were calculated to be 36% and 72% (Figure 3d). Comprehensive XPS, EDS, BET, TEM, UV-vis, and DLS characterization results confirm the stepwise formation from MPDA NPs to MPDA@MnO_2_ NPs to MPDA@MnO_2_@BA, validating the successful creation of MPDA@MnO_2_ NPs and drug incorporation.

### 3.2. Characterization of ChSMA-DA and Hydrogels

**Figure 4:**
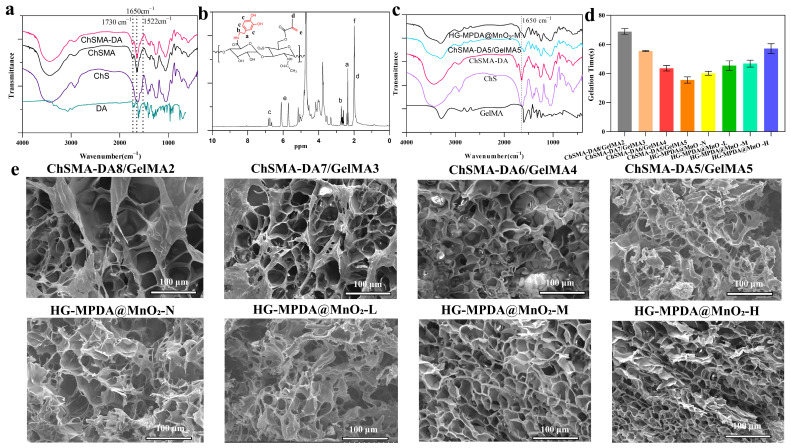
Characterization of ChSMA-DA and hydrogels. (**a**) FT-IR spectra of ChSMA-DA, (**b**) ^1^H NMR spectra of ChSMA-DA, ^1^H NMR (600 MHz, D_2_O): 2.3 ppm [a], 2.7 ppm [b], 6.8 ppm [c], 1.98 ppm [d], 6.10 ppm and 5.70 ppm [e], 2.00 ppm [f], (**c**) FT-IR spectra of hydrogels, (**d**) Gelation time of hydrogels, (**e**) SEM images of hydrogels.

The characteristic peaks of ChSMA chemical bonds were analyzed using FT-IR and ^1^H NMR spectroscopy. In the FT-IR spectra of ChSMA (Figure 4a), a distinct C=O stretching vibration peak, attributed to the ester bond and carbonyl group in the methylacryloyl moiety, was observed at approximately 1730 cm^−1^. Additionally, an unsaturated vibration signal corresponding to the C=C bond appeared near 1650 cm^−1^, indicating the presence of the carbon-carbon double bond in the methylacryloyl group. The observation of these peaks confirms the successful incorporation of the methylacryloyl group into the ChS molecular chain. In Figure 4b, the ^1^H NMR characterization of ChSMA revealed the methacrylate substitution ratio. Following the introduction of the methacryloyl group, characteristic peaks corresponding to hydrogen atoms on the carbon-carbon double bonds (e, C=CH_2_ peaks near 5.7 ppm and 6.1 ppm) and a methyl proton peak (d, -CH_3_ peak at 1.9 ppm) emerged in the NMR spectra. The degree of methacrylate substitution was calculated based on the ratio of the integral area of the methyl proton peak at 1.9 ppm for MAA to that of the methyl proton peak at 2.0 ppm (f) for ChS [43]. The grafting degree of MA was approximately 58%.

ChSMA-DA was characterized using FT-IR and ^1^H NMR spectroscopy. The FT-IR spectra (Figure 4a) reveal a distinctive peak at 1522 cm^−1^, corresponding to the ChSMA-DA, which is attributed to the C-N vibration of the dopamine aromatic skeleton. In Figure 4b, Peaks at 2.3 ppm, 2.7 ppm, and 6.8 ppm represent the -CH_2_ alkyl protons (a), (b), and the aromatic group protons (c) in dopamine, respectively, thereby confirming the successful synthesis of ChSMA-DA and indicating the effective grafting of dopamine [44]. The grafting degree of DA was 23%. The results from NMR and FT-IR analyses corroborate each other, demonstrating the successful synthesis of ChSMA-DA.

The hydrogels were prepared and characterized using FT-IR spectroscopy. In the FT-IR spectra of the hydrogels (Figure 4c), the characteristic peak corresponding to the carbon-carbon double bond of ChSMA-DA at 1650 cm^−1^ was absent, indicating that the carbon-carbon double bond of ChSMA-DA underwent polymerization with GelMA. The appearance of the hydrogels further confirmed the successful preparation of the hydrogels. The preparation conditions for the NPs-loaded hydrogel were consistent with those of a basic hydrogel, and the PDI was assessed in the precursor. PDI value of less than 0.2 confirmed the uniform dispersion of the NPs. Given the rapid gelation rate, it can be inferred that this uniform dispersion persisted following crosslinking.

### 3.3. Gelation Time of Hydrogels

Under specific light conditions, each precursor solution completed the sol-gel transformation in a short time. As shown in Figure 4d, the gelation time for the basic hydrogels was 68.86 ± 2.05 s, 55.55 ± 0.33 s, 43.58 ± 1.86 s, and 35.55 ± 2.13 s, respectively. As the proportion of GelMA increased, the gelation time of the system generally decreased. At higher ChSMA-DA content, the enhanced flexibility and hydrophilicity of the molecular chain resulted in a slight prolongation of the gelation time; however, it remained within the range suitable for in-situ gel application. In the NP-loaded hydrogels, the gelation time initially increased slowly before rising significantly. The gelation time for NP-loaded hydrogels was 40.14 ± 1.34 s, 45.43 ± 3.26 s, 46.82 ± 2.42 s, and 57.15 ± 3.30 s, respectively. This finding indicates that a high concentration (0.15% (*w*/*v*)) of MPDA@MnO_2_ NPs of NP-loaded hydrogels may partially shield UV light, occupy local cross-linking space, or interfere with the approach of molecular chain segments, thereby influencing the gelation ability. Hydrogels exhibit a relatively rapid gelation time. This material can effectively store the precursor liquid away from light. Upon injection, if it undergoes light-induced crosslinking once more, it can promptly gel and cure, thereby achieving the desired medical effects.

### 3.4. Microtopography of Freeze-Dried Hydrogels

SEM images (Figure 4e) reveal that the average pore size of the basic hydrogel decreases with a higher proportion of GelMA, leading to a denser network. A larger pore structure facilitates rapid liquid absorption and material exchange; however, this often compromises mechanical properties. Conversely, a smaller pore structure enhances mechanical stability and slows the drug diffusion rate. The incorporation of functional nanoparticles further reduced pore size, suggesting that the introduction of nanoparticles not only promoted the densification of the network skeleton but also compressed the pore space to some extent.

### 3.5. Swelling and Water Retention of Hydrogels

**Figure 5:**
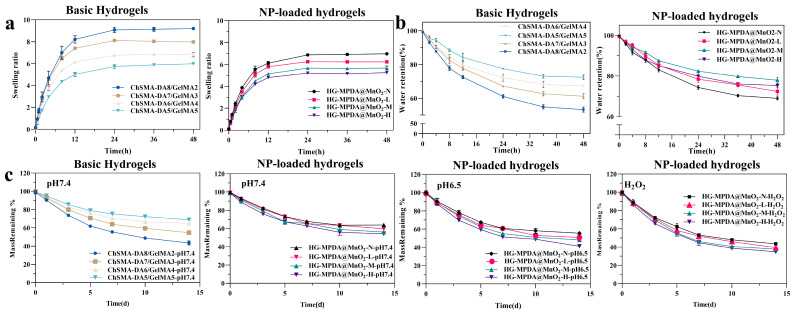
(**a**) Swelling ratio (SR) of hydrogels, (**b**) Water retention (WR) of hydrogels, (**c**) Mass remaining (MR) of hydrogels.

As shown in Figure 5a, the 24-h SR of the basic hydrogels with ChSMA-DA/GelMA ratios of 8:2, 7:3, 6:4, and 5:5 were 9.1 ± 0.2, 8.1 ± 0.1, 6.8 ± 0.1, and 5.7 ± 0.2, respectively. A decrease in the proportion of ChSMA-DA corresponded with a downward trend in the SR. This phenomenon can be attributed to the mechanism by which ChSMA-DA is rich in sulfate ions and catechol groups, both of which exhibit higher hydrophilicity and water absorption capacity. Additionally, sulfate ions and catechol groups introduce steric hindrance, resulting in decreased crosslinking density and an expanded hydrogel network space, which increases the SR and decreases the WR, and higher WR hydrogels suit exudative wounds. SEM observations confirmed that pore size significantly decreased with the reduction of ChSMA-DA proportion, aligning with the observed SR and WR trend. Although the 24-h SR of ChSMA-DA5/GelMA5 decreased to 6.8 ± 0.1, rheology results show notable improvements in network rigidity and dimensional stability. As a representative formulation reflecting a balance between performance attributes, the 24-h SR of HG-MPDA@MnO_2_-N was recorded at 6.9 ± 0.1, balancing both liquid absorption capacity and structural stability. Following the incorporation of increasing MPDA@MnO_2_ NPs, the 24-h SR of the NPs-loaded hydrogel further decreased to 6.3 ± 0.1, 5.7 ± 0.1, and 5.2 ± 0.1. The NPs might occupy the free volume within the network as physical packing phases, thereby promoting network densification and contributing to the reduction of the hydrogel’s SR.

The analysis of WR (Figure 5b) further confirmed the critical role of network structure in determining water retention capacity. In the 24 h dehydration test, the WR of the basic hydrogels was 61.08 ± 0.99%, 67.15 ± 0.49%, 72.14 ± 2.16%, and 77.50 ± 0.46% with decreasing ChSMA-DA content, demonstrating a significant negative correlation with SR (*R*^2^ = 0.99). Following the incorporation of MPDA@MnO_2_ NPs, the 24 h WR of the NP-loaded hydrogels were 74.35 ± 0.98%, 78.40 ± 1.55%, 82.42 ± 0.20%, and 79.87 ± 1.95%. Notably, HG-MPDA@MnO_2_-M achieved the highest WR at 82.42 ± 0.20%. The presence of MPDA NPs mesopores indicated that an optimal concentration of MPDA@MnO_2_ NPs could enhance the hydrogel’s WR. Conversely, the WR of HG-MPDA@MnO_2_-H slightly decreased to 79.87 ± 1.95%, suggesting that an excess of particles may disrupt water distribution within the pores. In conclusion, by optimizing nanoparticle loading, HG-MPDA@MnO_2_-M can maximize WR efficiency while maintaining structural integrity.

### 3.6. Degradation of Hydrogels

The degradation kinetics (Figure 5c) of hydrogels demonstrate a pronounced sensitivity to microenvironmental stimuli associated with pathological conditions, including weak acidity and elevated levels of ROS. This property underpins their potential for controlled release and in situ clearance at lesion sites, such as chronic wounds, tumors, or areas of inflammation. Specifically, under physiological conditions (PBS, pH 7.4), the MR of the basic hydrogels on the 14th day was measured at 46.8 ± 5.9%, 56.9 ± 3.9%, 66.1 ± 2.2%, and 67.4 ± 2.6% with decreasing ChSMA-DA content (increasing GelMA and cross-linking density), respectively. These results indicate a trend of decreasing MR with increasing ChSMA-DA content (and GelMA decreasing), affected by sulfate and catechol groups. A higher content of ChSMA-DA has a lower crosslinking density and a faster degradation rate, resulting in a lower MR. Lower MR is conducive to the full release of drugs, so we choose a sample with lower SR (higher WR) for NPs loading. Furthermore, the introduction of responsive NPs allows for the modulation of degradation behavior. The MR of the optimized HG-MPDA@MnO_2_-M hydrogels on the 14th day in three typical microenvironments was recorded at 56.4 ± 1.2% (PBS, pH 7.4), 46.0 ± 4.0% (PBS, pH 6.5), and 36.9 ± 1.5% (1 mmol·L^−1^ H_2_O_2_), with statistically significant differences observed (*p* < 0.001).

This phenomenon of accelerated gradient degradation suggests that an acidic environment (pH 6.5) can moderately enhance the disruption of the hydrogel network, whereas a ROS-rich environment induces more pronounced oxidation-triggered degradation. Research has demonstrated that the extent of responsive degradation correlates positively with the concentration of MPDA@MnO_2_ NPs. In conclusion, this hydrogel system not only exhibits adjustable baseline stability but also facilitates a synergistic recognition and amplification response to the characteristic signals of the lesion microenvironment (weak acid and high ROS) through the modulation of nano-component dosage. This capability enables selective degradation in pathological contexts such as chronic wounds and tumor microenvironments.

### 3.7. Rheological of Hydrogels

**Figure 6:**
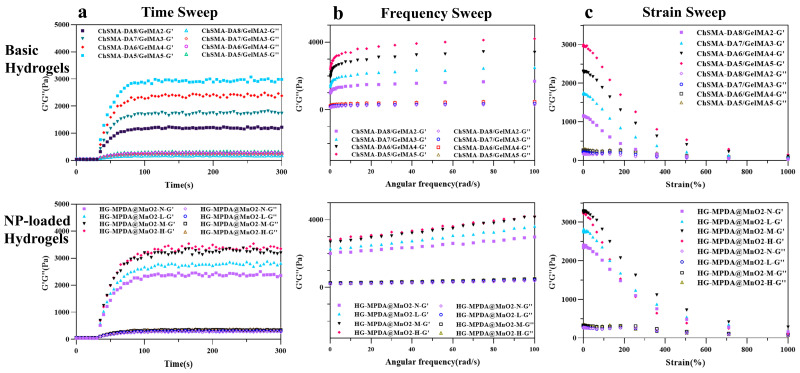
(**a**) Rheological properties of time sweep, (**b**) Rheological properties of frequency sweep, (**c**) Rheological properties of strain sweep.

As shown in Figure 6, hydrogels demonstrate controllable rheological properties. Their mechanical behavior integrates rapid photocrosslinking, structural stability, and shear adaptability, thereby fulfilling the demands of rapid prototyping and dynamic stability in biomedical applications. Time scanning (Figure 6a) demonstrated that following the initiation of crosslinking by 405 nm irradiation, the *G*′ of each hydrogel rapidly increased within tens of seconds, continuously exceeding *G*″, thereby achieving an efficient transition from liquid precursors to elastic solid states. Notably, the gelation point (*G*′ = *G*″) was reached in approximately 40 s, indicating that this system exhibits remarkable photo-initiated gel efficiency. In the frequency sweep (Figure 6b), *G*′ consistently surpassed the loss modulus *G*″ across the entire test frequency range (0.1–100 rad·s^−1^, selected to capture both terminal and plateau viscoelastic regimes typical of cross-linked hydrogel networks) with no intersection observed. This finding confirms that the three-dimensional network formed post-cross-linking possesses high uniformity and dynamic stability, and the substantial margin of G′ > G″ across physiologically relevant frequencies (0.1–100 rad·s^−1^) indicates a strong elastic dominance. This characteristic supports sustained shape fidelity and resistance to creep under prolonged static loading, which are essential attributes for maintaining conformal contact with wounds. During the strain sweep (Figure 6c), the linear viscoelastic region (LVR) was defined as the strain range over which both the G′ and G″ remained constant, and all hydrogel components exhibited well-defined LVRs extending up to 10% strain, confirming structural integrity of the network under small deformations, and better structural integrity with increasing content of GelMA and NPs. In the hydrogel network system, an increasing of GelMA and NPs correlates with a higher equilibrium state *G*′. In summary, this hydrogel system demonstrates synergistic optimization across three dimensions: gel dynamics, structural robustness, and mechanical programmability, thereby providing a rheological basis for its application in minimally invasive injection scenarios.

### 3.8. Injectability and Adhesion of Hydrogels

**Figure 7:**
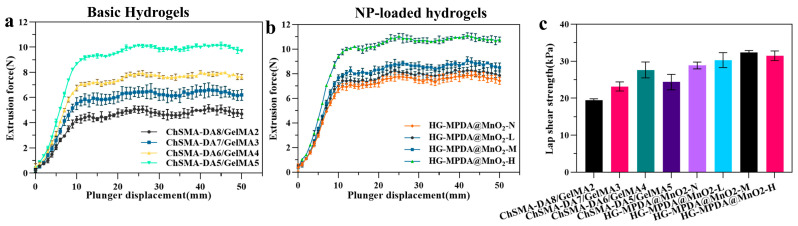
(**a**) Injectability properties of basic hydrogels, (**b**) Injectability properties of NP-loaded hydrogels, (**c**) Adhesion properties of hydrogels.

In injectability tests, the maximum injection forces for basic hydrogels (Figure 7a) with a ChSMA-DA/GelMA ratio of 8:2, 7:3, 6:4, and 5:5 were recorded as 5.20 ± 0.25 N, 6.70 ± 0.48 N, 8.06 ± 0.06 N, and 10.30 ± 0.08 N. These findings suggest that increased network density and viscosity are associated with heightened injection resistance. As shown in Figure 7b, HG-MPDA@MnO_2_-H exhibited the highest resistance; in contrast, HG-MPDA@MnO_2_-L and HG-MPDA@MnO_2_-M remained within an acceptable range for injection. Moderate rigidity preserves cohesive strength to resist debonding under physiological shear.

In adhesion tests (Figure 7c), the adhesion strengths of basic hydrogels (ChSMA-DA/GelMA ratio of 8:2, 7:3, 6:4, and 5:5) were measured at 19.49 ± 0.35, 23.17 ± 1.23, 27.66 ± 2.15, and 24.37 ± 2.07 kPa, respectively. Among these, ChSMA-DA6/GelMA4 exhibited the highest adhesion strength, indicating an optimal balance between the network’s cohesion strength and interfacial adhesion capability. In contrast, ChSMA-DA8/GelMA2, despite containing a greater proportion of hydrophilic chain segments, demonstrated relatively low adhesion strength due to its softer network and insufficient cohesion. Although ChSMA-DA5/GelMA5 featured the densest cross-linking, its adhesion performance was likely slightly inferior to that of ChSMA-DA6/GelMA4, attributed to a reduction in surface-accessible functional groups. Therefore, from an adhesion perspective, a ChSMA-DA/GelMA ratio of 6:4 or 5.5:4.5 approaches the optimal matrix state. The adhesion strengths of NP-loaded hydrogels (HG-MPDA@MnO_2_-N, HG-MPDA@MnO_2_-L, HG-MPDA@MnO_2_-M, and HG-MPDA@MnO_2_-H) were recorded at 28.87 ± 0.92, 30.32 ± 2.05, 32.38 ± 0.52, and 31.48 ± 1.31 kPa; these values significantly exceed typical adhesion thresholds reported for clinically used wound dressings (5–25 kPa), all of which have demonstrated effective in vivo wound closure and resistance to fluid washout. The introduction of an optimal concentration of nanoparticles enhances the cohesive structure and interfacial contact stability of the hydrogels. The HG-MPDA@MnO_2_-M exhibits the best performance, while the HG-MPDA@MnO_2_-H shows a slight decline. Excessively high particle concentrations can result in increased local rigidity or phase separation, potentially limiting the efficiency of interfacial adhesion. Taking into account both gelation time and WR, HG-MPDA@MnO_2_-M emerges as the most translationally viable candidate, delivering robust, clinically competitive adhesion without sacrificing injectability.

### 3.9. Photothermal of Hydrogels

**Figure 8:**
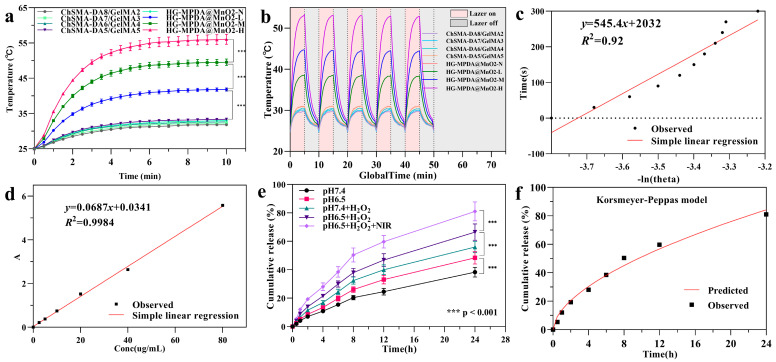
(**a**) Photothermal properties of hydrogels, (**b**) Over 5 irradiation cycles (808 nm, 1.0 W/cm^2^), (**c**) Fitting curves of the time logarithm of the temperature during the cooling process, (**d**) Standard curve of UV absorption for BA drugs, (**e**) Drug-load hydrogels’ BA release curves under different environment, (**f**) Fit of Korsmeyer-Peppas model.

The constructed hydrogel system, as illustrated in Figure 8a, demonstrates significant and controllable photothermal conversion capabilities. The underlying mechanism is attributed to the photothermal activity of dopamine groups in ChSMA-DA and the synergistic enhancement provided by MPDA@MnO_2_ NPs. Under irradiation with a 1.0 W·cm^−2^ near-infrared laser, all hydrogel samples exhibited an increase in temperature; however, systematic variations were observed in the degree of temperature elevation. The basic hydrogels without MPDA@MnO_2_ NPs recorded a temperature rise of approximately 5 °C. Although the temperature increase was slightly greater with a higher ChSMA-DA ratio (*p* < 0.001), the rise remained limited to less than 2 °C. In contrast, the introduction of MPDA@MnO_2_ NPs significantly amplified the photothermal response (*p* < 0.001). The NP-loaded hydrogels with MPDA@MnO_2_ NPs concentrations of 0, 0.05% (*w*/*v*), 0.10% (*w*/*v*), and 0.15% (*w*/*v*) reached temperatures of 33.01 °C, 41.82 °C, 49.54 °C, and 56.06 °C after achieving thermal equilibrium. This data illustrates a clear dose-dependent relationship with MPDA@MnO_2_ NPs, confirming that MPDA@MnO_2_ NPs is the critical functional component responsible for enhancing photothermal performance. PTT usually requires a temperature range of 45–50 °C; above 50 °C poses a risk of tissue damage, so the NPs-loaded hydrogel HG-MPDA@MnO_2_-M is most appropriate.

Furthermore, five consecutive laser “on-off” cycle tests revealed that each sample rapidly attained the corresponding steady-state temperature within each irradiation cycle. In Figure 8b, the fluctuation of the maximum temperature value in each cycle remained below ±0.5 °C, without exhibiting any attenuation trend. This finding indicates that the hydrogel system demonstrates remarkable structural stability and a reversible thermal response under repeated photothermal stimulation, and its photothermal conversion efficiency remains unaffected by material fatigue or component degradation. In addition, the thermal time constant (*τ*s) was calculated to be 545.4 (Figure 8c), and the photothermal conversion efficiency (η) was calculated to be 22.7% from cooling curve (Figure 8b).

### 3.10. In Vitro Drug Loading and Release of Hydrogels

Figure 8d illustrates the standard curve for BA, represented by the equation *y* = 0.0687*x* + 0.0341, *R*^2^ = 0.9984. Following the incorporation of BA into the hydrogel, a multi-stimulation-responsive release behavior was observed (Figure 8e). The hydrogel exhibited synergistic responsiveness to pH, ROS, and NIR stimulation. As a response coating, MnO_2_ decomposes into Mn^2+^ under acidic conditions at pH 6.5, leading to material degradation and drug release. Additionally, MnO_2_ can catalyze the decomposition of H_2_ and O_2_ to generate O_2_, facilitating the hydrogel’s ability to eliminate ROS. Furthermore, both MPDA@MnO_2_ NPs and ChSMA-DA are abundant in catechol structures, which confer photothermal properties to the hydrogel. Under weak acid and ROS conditions, both will accelerate the degradation of MnO_2_ and achieve drug release. The photothermal effect will cause the solution to heat up and also accelerate the temperature-accelerated diffusion of the drug. A single stimulus elicited only a limited release; the simultaneous application of multiple stimuli significantly enhanced the kinetics of drug release. This approach enables a high degree of adaptability and precise external regulation of microenvironments, such as those found in chronic wounds and tumors. The 24 h cumulative release percentage of the HG-MPDA@MnO_2_-M hydrogel exhibited a strictly monotonically increasing relationship under various microenvironmental simulation conditions: pH 7.4 (38.31%) < pH 6.5 (48.39%, increasing 10.08% vs. pH 7.4) < pH 7.4 + H_2_O_2_ (55.93%, increasing 17.62% by H_2_O_2_) < pH 6.5 + H_2_O_2_ (66.46%, increasing 18.07% by H_2_O_2_) < pH 6.5 + H_2_O_2_ + NIR (81.06%, increasing 14.60% by NIR). It was confirmed that the weakly acidic pH not only accelerated the release kinetics but also worked in conjunction with ROS to produce an additive effect. On this basis, NIR irradiation (808 nm, 1.0 W/cm^2^, for 5 min) was subsequently applied. The photosensitive components within the hydrogel induced photothermal effects, leading to a localized increase in temperature that further enhanced chain segment movement and pore expansion. This resulted in a 24 h cumulative release percentage of 81.06%, significantly improving the cumulative release percentage of BA. This gradient release behavior not only quantitatively validated the effectiveness of the multi-stimulus synergy mechanism but also closely aligned with the typical environmental characteristics associated with chronic wounds and solid tumor microenvironments, including reductions in interstitial pH and increases in endogenous ROS levels. Furthermore, the system enables spatiotemporal controllable “on-demand triggering” through exogenous NIR light, thereby providing experimental evidence for the targeted and synergistic delivery of baicalin.

### 3.11. Kinetic Modeling of Baicalin Release

The drug BA release kinetics simulations of HG-MPDA@MnO_2_-M hydrogel in three co-stimulatory environments are shown in Table 2. It was confirmed that the Korsmeyer-Peppas model (*R*^2^ = 0.9877, Figure 8f) exhibited the highest regression coefficient, indicating it is the best-fitting model among all evaluated. The *n* value of the Korsmeyer-Peppas model, *n* = 0.5705 (0.45 < *n* < 0.89), suggests that the drug release mechanism can be characterized as “anomalous transport”. This implies that the drug BA release is mainly contributed through a synergistic interplay of diffusion and polymer swelling.

## 4. Conclusions

In this study, nanotechnology was integrated with advanced PTT and hydrogels to develop an intelligent, responsive hydrogel tailored for chronic wound treatment. The synthesized hydrogel, designated HG-MPDA@MnO_2_-M, exhibited optimal overall performance, demonstrating favorable injectability, adhesive properties, and photothermal conversion efficiency. Additionally, it was capable of concurrently sensing the weakly acidic and high ROS pathological microenvironment within the tissue, along with externally applied NIR light, thereby facilitating multifactorial synergistic regulation. Several limitations merit consideration for future translation. The in vivo stability of MPDA@MnO_2_ NPs, specifically their degradation kinetics, Mn^2+^ ion release profile, and potential long-term accumulation, requires further pharmacokinetic and biodistribution studies. Although NIR-triggered release and photothermal modulation show promise in preclinical models, clinical translation will depend on the optimization of light delivery parameters. In summary, this work demonstrates that the NIR-responsive, microenvironment-sensing HG-MPDA@MnO_2_-M hydrogel facilitates spatiotemporally controlled BA release, with anticipated applications in chronic wounds for intelligent, stimuli-adaptive therapeutics.

## Figures and Tables

**Table 1:** The composition of hydrogels (10 mL).

Group	Samples	ChSMA-DA	GelMA	MPDA@MnO_2_ NPs	LAP
Basic hydrogels	ChSMA-DA8/GelMA2	0.80 g	0.20 g	N/A	25 mg
	ChSMA-DA7/GelMA3	0.70 g	0.30 g	N/A	25 mg
	ChSMA-DA6/GelMA4	0.60 g	0.40 g	N/A	25 mg
	ChSMA-DA5/GelMA5	0.50 g	0.50 g	N/A	25 mg
NP-loaded hydrogels	HG-MPDA@MnO_2_-N	0.55 g	0.45 g	N/A	25 mg
	HG-MPDA@MnO_2_-L	0.55 g	0.45 g	5 mg	25 mg
	HG-MPDA@MnO_2_-M	0.55 g	0.45 g	10 mg	25 mg
	HG-MPDA@MnO_2_-H	0.55 g	0.45 g	20 mg	25 mg

**Table 2:** Kinetic modeling of baicalin release in pH 6.5 + H_2_O_2_ + NIR.

Modle	Best-Fit Values of Parameter		*R* ^2^
	Parameter	Best-Fit Values	
Zero-order	k_0_	4.1141	0.7879
First-order	k_1_	0.0806	0.9875
Higuchi	k_H_	16.3435	0.9790
Hixson–Crowell	k_HC_	0.0230	0.9642
Korsmeyer–Peppas	k_KP_	13.7430	0.9877
	*n*	0.5705	
Baker–Lonsdale	k_BL_	0.0061	0.9533

## Data Availability

The original contributions presented in this study are included in the article/Supplementary Material. Further inquiries can be directed to the corresponding authors.

## Supplementary Materials

The following supporting information can be downloaded at: https://www.mdpi.com/article/10.3390/polym18111351/s1, NMR Spectra of ChSMA-DA.

## Abbreviations

The following abbreviations are used in this manuscript:

ChS	Chondroitin sulfate
Gel	Gelatin
DA	Dopamine
MAA	Methacrylic anhydride
EDC	1-(3-Dimethylaminopropyl)-3-ethylcarbodiimide
NHS	*N*-Hydroxy succinimide
MPDA	Mesoporous polydopamine NPs
KMnO_4_	Potassium permanganate
FA	Formamide
MnO_2_	Manganese dioxide
NPs	Nanoparticles
LAP	Lithium acylphosphonate
SR	Swelling ratio
WR	Water retention
DLS	Dynamic light scattering
BET	Brunauer-Emmet-Teller
SEM	Scanning electron microscopy
TEM	Transmission electron microscopy
PBS	Phosphate buffered saline
BA	Baicalin
PTT	Photothermal therapy
MR	Mass remaining
