# Supplementary figures and images for: Chondroitin Sulfate-Based MPDA@MnO2 Nanocomposite Hydrogels: A Smart Drug Delivery System with pH/ROS Responsiveness and Photothermal-Enhanced Therapeutic Effects
Source: Polymers (Basel). 2026 May 29;18(11):1351. doi: 10.3390/polym18111351 (PMC13259469; doi:10.3390/polym18111351)

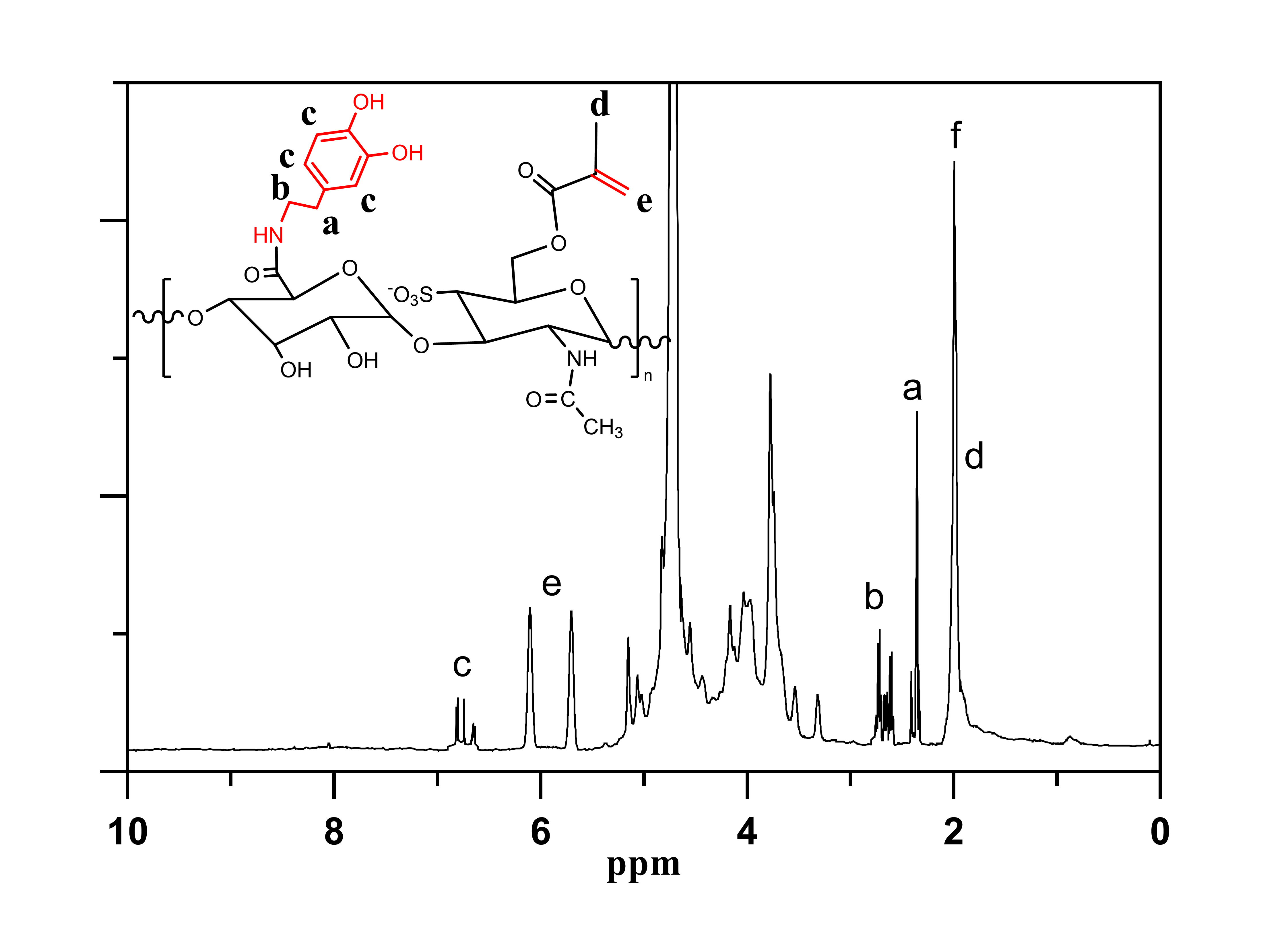

Supplement: Supplementary file 1 [file polymers-18-01351-s001.zip › polymers-4335257-supplementary.tif]
